# Immunological aspects of radiotherapy

**DOI:** 10.1186/1748-717X-9-185

**Published:** 2014-08-20

**Authors:** Heike Scheithauer, Claus Belka, Kirsten Lauber, Udo S Gaipl

**Affiliations:** Department of Radiotherapy and Radiation Oncology, Ludwig Maximilian University Munich, D-81377 Munich, Germany; Department of Radiation Oncology, Radiation Immunobiology, University Hospital Erlangen, Friedrich-Alexander-Universität, Erlangen-Nürnberg, Germany

The major objective of radiotherapy (RT) in the context of cancer treatment is the achievement of local tumor control. Traditionally, this is considered to be mediated by the induction of DNA damage, resulting in tumor cell death and abrogated clonogenic survival [1]. However, it has become more and more evident that distinct irradiation regimes as well as selected doses of ionizing radiation, and particularly the combination with immunotherapeutic approaches can induce or modulate systemic immune responses, which contribute to tumor control or inflammatory side effects, respectively. Locally administered RT can instigate systemic, abscopal, or 'out-of-field' effects, and meanwhile it is well acknowledged that DNA damage responses and immunological events, including anti-tumor immune mechanisms and inflammatory reactions are interconnected. The Special Topic *Immunological aspects of radiotherapy* aims to introduce radiation oncologists and researchers in the field of molecular and cellular oncology to the manifold aspects of how RT impacts on immune modulation, and how the combination with targeted therapies and selected immunotherapeutic strategies can result in improved local and systemic tumor control via the stimulation of anti-tumor immune responses. Focus is set on the immunological effects of different irradiation regimes and doses, synergistic effects between RT and immunotherapy with natural killer cells or mRNA-based vaccines, and finally on immunological normal tissue reactions.

The interaction of monocytes and dying breast cancer cells that have been subjected to different irradiation regimes is addressed in the study by Hennel et al. [2]. The authors characterize the type and the extent of cell death induced by fractionated and ablative radiotherapeutic regimes as well as the impact on the release of danger signals and monocyte attraction factors by dying breast cancer cells. In essence, they describe that the irradiation regime as well as the p53 and hormone receptor status govern the cell death response and subsequent monocyte recruitment. Whereas fast proliferating, p53 mutant, hormone receptor negative breast cancer cells predominantly underwent primary necrosis upon ablative irradiation at a single dose of 20 Gy, p53 wildtype breast cancer cells revealed a multi-faceted response of apoptosis, primary/secondary necrosis, and senescence. Compared to fractionated irradiation at daily doses of 2 Gy, a much stronger cellular response in terms of apoptosis, necrosis and senescence induction was achieved by ablative irradiation. Importantly, necrotically dying, p53 mutant, hormone receptor negative breast cancer cells released apyrase-sensitive nucleotides - well-known danger signals, which stimulated monocyte chemokinesis. In p53 wildtype, hormone receptor positive cells this was hampered by the upregulation of the surface ectonucleotidase CD39. Given that the intra-tumoral recruitment of monocytes, their differentiation into antigen-presenting cells, the capture of tumor antigens, and the subsequent trafficking into tumor-draining lymph nodes constitute initial and essential steps for the priming of adaptive anti-tumor immune responses [3], the authors conclude that especially for fast proliferating, hormone receptor negative, p53 mutant breast cancer ablative RT might be beneficial. Future studies have to clarify, whether the cascade of targeted necrosis induction, nucleotide release, and monocyte recruitment indeed can trigger the priming of adaptive anti-tumor immunity in the context of ablative radiotherapy [4].

The type of cell death response and concomitant danger signal release is also in the focus of the study by Rubner et al. [5]. Using glioblastoma cell lines with different p53 and O6-methylguanine DNA methyltransferase (MGMT) expression status, the authors examine the induction of glioblastoma cell death upon fractionated RT at daily doses of 2 Gy alone or in combination with clinically relevant concentrations of temozolomide (TMZ) and/or the histone deacetylase (HDAC) inhibitor valproic acid (VPA). As to be expected, p53 mutant, MGMT expressing glioblastoma cells were more resistant to fractionated RT +/- TMZ or VPA treatment and showed increased clonogenic survival compared to p53 wildtype, MGMT negative cells, since they presumably exhibit improved MGMT-mediated DNA damage repair and compromised p53-dependent cell death and senescence mechanisms [6]. Along the same lines, TMZ-induced G2 cell cycle arrest was only observed in MGMT negative cells with wildtype p53, and RT-induced G2 cell cycle arrest was much more pronounced than in p53 mutant, MGMT positive cell lines. Importantly, fractionated RT was the main stimulus for apoptosis as well as necrosis induction with concomitant release of the danger signals heat-shock protein 70 (Hsp70) and high-mobility group protein B1 (HMGB1) in p53 mutant, MGMT expressing cells. Correspondingly, the authors conclude that especially in p53 mutant, MGMT positive glioblastoma fractionated RT and not chemotherapy with TMZ or VPA governs cell death induction and release of danger signals. Both might be relevant for shaping an immunogenic tumor microenvironment necessary for the induction of systemic anti-tumor immunity, and future research has to focus on how RT might contribute to the success of multimodal immunotherapeutic approaches for glioblastoma multiforme [7].

The danger signal Hsp70 has been shown to activate dendritic cells (DC) as well as natural killer (NK) cells, and tumor cells are known to upregulate the expression of this chaperone, since they experience a sort of constitutive proteotoxic stress due to an overall increase in protein synthesis and the overexpression of various mutant oncoproteins [8]. Hsp70 might also be exposed on the tumor cell surface, and thus appears to be a promising tumor biomarker as well as a potential target for tumor therapy. Besides monitoring local responses by analyzing Hsp70 in tumor biopsies, systemic effects might be followed up by determining the serum concentration of released Hsp70. This issue is addressed by the study of Gehrmann et al. in the context of adjuvant RT in patients with head and neck squamous cell carcinoma (SCCHN) [9]. In 22 out of 23 single cell suspensions of tumor biopsies, Hsp70 membrane expression was increased compared to normal tissue cells. Tumors with low and high Hsp70 expression levels were identified, and the serum concentrations of Hsp70 before tumor resection were elevated in all patients compared to healthy donors. During adjuvant RT, serum Hsp70 levels increased up to 6 weeks after tumor excision and declined afterwards to levels similar to those before RT. With a timely delay, elevated anti-Hsp70 antibody titers were observed in patients' sera. Importantly, Hsp70 and anti-Hsp70 antibody serum levels correlated with the tumor volume before therapy. Analyses of activation markers on peripheral blood NK cells revealed an increase in the expression densities of NKG2D, but not CD56, CD94, nor NKp44 throughout the monitoring period. In summary, the authors propose the serum level of Hsp70 as a biomarker for tumor detection and RT monitoring in SCCHN, whose applicability has to be evaluated in further studies [10].

Son et al. addressed the question if and how RT alone or in combination with HDAC inhibition alters the expression of NKG2D ligands in non-small cell lung cancer (NSCLC) cell lines. NKG2D is an activating NK cell receptor, and the induction of NKG2D ligands in cancer cells is known to be regulated by histone acetylation and - at least in part - by the Atm (Ataxia Telangiectasia Mutated protein)/Atr (Ataxia Telangiectasia and Rad3-related protein) pathway, underscoring once more the connection between DNA damage responses and immune reactions [11]. While HDAC inhibition increased the expression of several NKG2D ligands, including MICA and ULBP3, on the mRNA as well as on the surface protein level, RT at a single dose of 8, 16, or 24 Gy did so only on the surface protein level. Importantly, the combination of RT and HDAC inhibition stimulated a supra-additive elevation of NKG2D ligands on the tumor cell surface, which was paralleled by a highly increased sensitivity towards NK cell-mediated lysis. HDAC inhibitor-dependent induction of NKG2D ligand mRNA was not affected by Atm/Atr inhibition, but RT-induced upregulation of NKG2D surface expression was significantly impaired. The authors conclude that the expression of NKG2D ligands is orchestrated on multiple levels, and that the synergistic upregulation observed by the combination of RT with HDAC inhibition might be utilized for the improvement of NK cell-based therapies in NSCLC with functional Atm/Atr signaling [12].

An *in vivo* evaluation of the synergism between RT and mRNA-based vaccination for the treatment of established tumors is presented in the study by Fotin-Mleczek et al. [13]. Vaccination strategies alone commonly fail to eradicate large tumors due to the numerous immune evasion mechanisms established tumors have acquired [14]. In this regard, RT is a highly promising 'partner' for combined modality approaches, since it locally destroys the tumor, can induce an immunostimulatory tumor microenvironment, and systemically spares the immune system (in contrast to chemotherapy). Fotin-Mleczek and coworkers employed this concept for RT +/- vaccination with OVA or EGFR mRNA of heterotopically transplanted, highly immunogenic E.G7-ovalbumin(OVA) lymphoma or poorly immunogenic Lewis lung carcinoma (LLC), respectively. In both model systems combined radio-immunotherapy revealed strong synergistic anti-tumor effects as displayed by potently delayed tumor growth or even complete tumor eradication, while the single treatments had only moderate or hardly detectable effects. Of note, completely responding E.G7-OVA lymphoma carrying mice even survived subsequent re-challenge with parental, OVA-negative EL-4 cells suggesting that the combined treatment induced immunological memory and epitope spreading. Transcriptome analyses of the E.G7-OVA lymphoma model revealed a unique gene signature in the radio-immunotherapy group involving downregulation of tumor associated genes and upregulation of genes mediating tumor suppression. Characterization of infiltrating immune cells in the LLC model showed that the combined treatment specifically stimulated an increase in tumor infiltrating CD4+ and CD8+ T cells as well as NKT cells, which was not detected in the single treatment groups. The authors conclude that local RT is capable of sculpting an immunogenic tumor microenvironment, which renders even poorly immunogenic tumors susceptible for mRNA-based vaccination leading to long lasting anti-tumor immune memory and even epitope spreading. Further mechanistic analyses are required to elucidate the underlying mechanisms, but a comparable form of epitope drift has already been observed in clinical phase II/III trials with a poxviral vaccine encoding PSA in combination with RT for prostate cancer [15].

Apart from modulating intended, anti-tumor-directed immune effects, RT can stimulate unwanted, immune cell-driven, adverse effects. Pneumonitis and lung fibrosis are examples of such dose-limiting side effects, which are observed in the context of thorax RT, and whose underlying mechanisms are so far barely understood [16]. Wirsdörfer et al. examined the presence and/or infiltration of distinct immune cell subsets in different organs upon thorax irradiation of C57BL/6 mice [17]. The authors report that irradiation-induced pneumonitis was associated with a characteristic time course of local and systemic changes within the T cell compartment. Upon single thorax irradiation at 15 Gy, a transient decrease in systemic CD4+ T cell counts and a long-lasting decrease in CD8+ T cells within peripheral lymphoid organs were observed. Furthermore, the early phase of irradiation-induced pneumonitis was paralleled by a local (lung) and systemic (spleen, cervical lymph nodes), but transient accumulation of CD4 + FoxP3+ regulatory T cells (Treg). These Treg exhibited immunosuppressive function as could be expected from the observed surface expression of immunosuppressive CD73, CTLA-4, and CD103. The authors speculate that the accumulation of Treg during early pneumonitis is due to their increased survival after irradiation compared to effector T cells. Treg might contribute to the control of irradiation-induced pneumonitis and limit inflammation-associated lung damage. Hence, it remains to be elucidated, whether irradiation-induced pneumonitis exacerbates if Treg function is impaired, and whether this can be therapeutically addressed in the future.

RT can also exert immunosuppressive functions. This is particularly observed in the low dose range, and is exploited therapeutically in the context of acute and chronic inflammatory diseases [18]. Accumulating evidence suggests that modulation of endothelial cells (EC), lymphocytes, macrophages, and granulocytes is key for the anti-inflammatory effects of low dose radiotherapy (LD-RT). Interestingly, the observed immunomodulatory consequences of LD-RT display a non-linear dose response relationship, which is a central characteristic of bystander effects induced by ionizing irradiation and which is attributed to the involvement of multiple molecular mechanisms that may be instigated at various threshold doses. The study presented by Large et al. addresses the connection between dampening effects of LD-RT on activated EC, production of reactive oxygen species (ROS), and DNA damage repair [19]. The authors report that one day after irradiation of TNF-stimulated EC at 0.5 Gy, but not at 0.3 nor 0.7 Gy, increased numbers of residual γH2AX foci were detected. Irrespective of TNF stimulation, this was accompanied by reduced expression levels and enzymatic activity of superoxide dismutase (SOD) with concomitantly increased ROS levels - again only upon irradiation at 0.5 Gy. Since previous studies described a local dose maximum for activation of the transcription factor NF-κB after irradiation of EC at 0.5 Gy, the authors speculate that increased DNA double strand breaks as well as augmented NF-κB activation both result from elevated ROS levels after irradiation at 0.5 Gy. In summary, these results suggest that mechanisms of RT-induced DNA damage response and immune modulation are interconnected and follow a non-linear, discontinuous dose response relationship, at least in the low and moderate dose range.

The preclinical *in vitro* and *in vivo* studies presented in our Special Topic *Immunological aspects of radiotherapy* convincingly reveal that RT can contribute to enhance the immunogenicity of tumor cells as well as their *in vivo* microenvironment, and that RT can successfully be combined with selected immunotherapeutic approaches for the attendance of glioblastoma, SCCHN, NSCLC, lymphoma, and LLC model systems. In turn, cells of the innate immune system (like monocytes, macrophages, and NK cells), those connecting innate and adaptive immunity (like DC and NKT cells), and those of the adaptive immune system (like CD4+ and CD8+ T cells) contribute to the outcome of RT and/or radio-immunotherapy. Seminal evidence suggests that DNA damage responses are linked to innate as well as adaptive immune mechanisms. Apart from the intended effects in terms of tumor cell death induction and the stimulation of anti-tumor immunity this interconnection might also influence the onset of adverse RT side effects, including irradiation-induced pneumonitis, as well as their subsequent resolution.

A crucial issue in the context of RT-stimulated immunological effects is the dose-response relationship, which appears to follow a discontinuous pattern, particularly when low dose irradiation is utilized to attenuate acute or chronic inflammation. In the higher dose range, fractionated RT regimes clearly differ from ablative single dose regimes with regard to the induction of tumor cell death and the stimulation of immune cell recruitment. Additionally, the p53 status, the hormone receptor status, functional Atm/Atr signaling, and presumably many more - *prima vista* unrelated - characteristics of tumor cells impact on the immunological properties of irradiated tumor cells, including the type of tumor cell death they undergo, the induction of NKG2D ligands, and the stimulation of monocyte recruitment.

Hence, the major challenges for the future are to define the optimal dose of RT together with optimized fractionation regimes and to design choreography and chronology of combined modality strategies with selected immunotherapeutic approaches carefully. By doing so, we might be able to advance RT towards optimal local tumor control with concomitant stimulation of long-lasting, systemic anti-tumor immunity and simultaneous avoidance of unwanted side effects. In this regard, RT appears to be 'a perfect match' for immunotherapy and - apart from its prominent role in DNA damage induction - should be considered as *in situ* inducer of immunogenic tumor cells. Finally, we would like to thank all authors, who have contributed to this Special Topic on emerging basic and preclinical research as well as clinical perspectives of *immunological aspects of radiotherapy*.

## References

[1] Orth M, Lauber K, Niyazi M, Friedl AA, Li M, Maihofer C, Schuttrumpf L, Ernst A, Niemoller OM, Belka C (2014). Current concepts in clinical radiation oncology. Radiat Environ Biophys.

[2] Hennel R, Brix N, Seidl K, Ernst A, Scheithauer H, Belka C, Lauber K (2014). Release of monocyte migration signals by breast cancer cell lines after ablative and fractionated gamma-irradiation. Radiat Oncol.

[3] Kroemer G, Galluzzi L, Kepp O, Zitvogel L (2013). Immunogenic cell death in cancer therapy. Annu Rev Immunol.

[4] Lauber K, Ernst A, Orth M, Herrmann M, Belka C (2012). Dying cell clearance and its impact on the outcome of tumor radiotherapy. Front Oncol.

[5] Rubner Y, Muth C, Strnad A, Derer A, Sieber R, Buslei R, Frey B, Fietkau R, Gaipl US (2014). Fractionated radiotherapy is the main stimulus for the induction of cell death and of Hsp70 release of p53 mutated glioblastoma cell lines. Radiat Oncol.

[6] Tabatabai G, Stupp R, van den Bent MJ, Hegi ME, Tonn JC, Wick W, Weller M (2010). Molecular diagnostics of gliomas: the clinical perspective. Acta Neuropathol.

[7] Hegde M, Bielamowicz KJ, Ahmed N (2014). Novel approaches and mechanisms of immunotherapy for glioblastoma. Discovery Med.

[8] Murphy ME (2013). The HSP70 family and cancer. Carcinogenesis.

[9] Gehrmann M, Specht HM, Bayer C, Brandstetter M, Chizzali B, Duma M, Breuninger S, Hube K, Lehnerer S, van Phi V, Sage E, Schmid TE, Sedelmayr M, Schilling D, Sievert W, Stangl S, Multhoff G (2014). Hsp70–a biomarker for tumor detection and monitoring of outcome of radiation therapy in patients with squamous cell carcinoma of the head and neck. Radiat Oncol.

[10] Langer CJ (2012). Exploring biomarkers in head and neck cancer. Cancer.

[11] Hoglund P: **DNA damage and tumor surveillance: one trigger for two pathways.** *Sci STKE* 2006, (317)**:**2. doi:10.1126/stke.3172006pe2 10.1126/stke.3172006pe2 16403940

[12] Rosental B, Appel MY, Yossef R, Hadad U, Brusilovsky M, Porgador A (2012). The effect of chemotherapy/radiotherapy on cancerous pattern recognition by NK cells. Curr Med Chem.

[13] Fotin-Mleczek M, Zanzinger K, Heidenreich R, Lorenz C, Kowalczyk A, Kallen K-J, Huber SM (2014). mRNA-based vaccines synergize with radiation therapy to eradicate established tumors. Radiat Oncol.

[14] Aldrich JF, Lowe DB, Shearer MH, Winn RE, Jumper CA, Kennedy RC (2010). Vaccines and immunotherapeutics for the treatment of malignant disease. Clinical Dev Immunol.

[15] Gulley JL, Madan RA, Tsang KY, Jochems C, Marte JL, Farsaci B, Tucker JA, Hodge JW, Liewehr DJ, Steinberg SM, Heery CR, Schlom J (2014). Immune impact induced by PROSTVAC (PSA-TRICOM), a therapeutic vaccine for prostate cancer. Cancer Immunol Res.

[16] Ding J, Wu Z, Crider BP, Ma Y, Li X, Slaughter C, Gong L, Xie XS (2000). Identification and functional expression of four isoforms of ATPase II, the putative aminophospholipid translocase. Effect of isoform variation on the ATPase activity and phospholipid specificity. J Biol Chem.

[17] Wirsdorfer F, Cappuccini F, Niazman M, de Leve S, Westendorf AM, Ludemann L, Stuschke M, Jendrossek V (2014). Thorax irradiation triggers a local and systemic accumulation of immunosuppressive CD4+ FoxP3+ regulatory T cells. Radiat Oncol.

[18] Rodel F, Frey B, Gaipl U, Keilholz L, Fournier C, Manda K, Schollnberger H, Hildebrandt G, Rodel C (2012). Modulation of inflammatory immune reactions by low-dose ionizing radiation: molecular mechanisms and clinical application. Curr Med Chem.

[19] Large M, Reichert S, Hehlgans S, Fournier C, Rodel C, Rodel F (2014). A non-linear detection of phospho-histone H2AX in EA.hy926 endothelial cells following low-dose X-irradiation is modulated by reactive oxygen species. Radiat Oncol.

